# Common Statin Intolerance Variants in *ABCB1* and *LILRB5* Show Synergistic Effects on Statin Response: An Observational Study Using Electronic Health Records

**DOI:** 10.3389/fgene.2021.713181

**Published:** 2021-10-01

**Authors:** Alaa’ Lutfi Melhem, Mehul Kumar Chourasia, Margherita Bigossi, Cyrielle Maroteau, Alasdair Taylor, Roberto Pola, Adem Y. Dawed, Aleksi Tornio, Colin N. A. Palmer, Moneeza K. Siddiqui

**Affiliations:** ^1^Division of Population Health & Genomics, Pat McPherson Centre for Pharmacogenetics & Pharmacogenomics, School of Medicine, Ninewells Hospital & Medical School, University of Dundee, Dundee, United Kingdom; ^2^Section of Internal Medicine and Thromboembolic Diseases, Department of Medicine, Fondazione Policlinico Universitario A. Gemelli IRCCS, Rome, Italy; ^3^Integrative Physiology and Pharmacology Unit, Institute of Biomedicine, University of Turku, Turku, Finland

**Keywords:** pharmacogenomics, non-HDL-cholesterol, *ABCB1*, *LILRB5*, statins, hyperlipidaemia

## Abstract

**Background:** Statin intolerance impacts approximately 10% of statin users, with side effects ranging from mild myalgia to extreme intolerance resulting in myopathy and rhabdomyolysis. Statin intolerance results in poor adherence to therapy and can impact statin efficacy. Many genetic variants are associated with statin intolerance. The effect of these variants on statin efficacy has not been systematically explored.

**Methods:** Using longitudinal electronic health records and genetic biobank data from Tayside, Scotland, we examined the effect of seven genetic variants with previously reported associations with simvastatin or atorvastatin intolerance on the outcome of statin response. Statin response was measured by the reduction achieved when comparing pre- and post-statin non-high-density lipoprotein-cholesterol (non-HDL-C). Post-treatment statin response was limited to non-HDL-C measured within 6months of therapy initiation. Univariate and multivariable linear regression models were used to assess the main and adjusted effect of the variants on statin efficacy.

**Results:** Around 9,401 statin users met study inclusion criteria, of whom 8,843 were first prescribed simvastatin or atorvastatin. The average difference in post-treatment compared to pre-treatment non-HDL-cholesterol was 1.45 (±1.04) mmol/L. In adjusted analyses, only two variants, one in the gene ATP-binding cassette transporter B1 (*ABCB1*; rs1045642), and one in leukocyte immunoglobulin like receptor B5 (*LILRB5*; rs12975366), were associated with statin efficacy. In *ABCB1*, homozygous carriers of the C allele at rs1045642 had 0.06mmol/L better absolute reduction in non-HDL-cholesterol than carriers of the T allele (95% CI: 0.01, 0.1). In *LILRB5* (rs12975366), carriers of the C allele had 0.04mmol/L better absolute reduction compared to those homozygous for the T allele (95% CI: 0.004, 0.08). When combined into a two-variant risk score, individuals with both the rs1045642-CC genotype and the rs12975366-TC or CC genotype had a 0.11mmol/L greater absolute reduction in non-HDL-cholesterol compared to those with rs1045642-TC or TT genotype and the rs12975366-TT genotype (95% CI: 0.05, 0.16; *p*<0.001).

**Conclusion:** We report two genetic variants for statin adverse drug reactions (ADRs) that are associated with statin efficacy. While the *ABCB1* variant has been shown to have an association with statin pharmacokinetics, no similar evidence for *LILRB5* has been reported. These findings highlight the value of genetic testing to deliver precision therapeutics to statin users.

## Introduction

Statins, or 3-hydroxy-3-methylglutaryl coenzyme A reductase inhibitors, are the most commonly prescribed cholesterol-lowering therapy (Ward et al., 2019). Statin at maximum doses can reduce low density lipoprotein-cholesterol (LDL-C) levels by 50% (Schachter, 2005; Mangravite et al., 2006; Taylor and Thompson, 2018; Newman et al., 2019). Large randomized clinical trials have reported a 20–30% reduction of cardiovascular diseases (CVD) among statin users (Baigent et al., 2005; Mangravite et al., 2006; Mihaylova et al., 2012).

However, there are interindividual differences in response to statin treatment: both in the ability of statins to reduce the LDL-C levels and in observed statin-related adverse drug reactions (ADRs) (Turner and Pirmohamed, 2019). It is estimated that 30% of statin users cease therapy by the end of the first year of treatment. Approximately 50% of patients at high risk of developing CVD discontinue taking their statins (Pedro-Botet et al., 2019; Bair et al., 2020). Among those who withdraw from treatment, about 1–7% discontinue taking statins due to ADRs (Vermes and Vermes, 2004; Oh et al., 2007; Donnelly et al., 2011).

Stain-induced ADRs can range from complaints of muscle pain referred to as myalgia to the more severe cases of myopathy, and finally, in extreme cases, can result in rhabdomyolysis (Alfirevic et al., 2014; Selva-O’Callaghan et al., 2018; Newman et al., 2019). Almost 60% of adults who discontinue statin therapy report muscle pain as the major cause of non-adherence (Pedro-Botet et al., 2019). It is understood that myalgia, whether or not associated with an elevation in the creatine kinase (CK) level, is the most common statin-induced ADR and is included in definitions of statin intolerance (Bair et al., 2020). The risk of ADR is greater during the first year of therapy (Armitage et al., 2010) and can be exacerbated by increases in statin dose, interacting concomitant medications, advanced age, or comorbidities (Newman et al., 2019). The exact prevalence of statin intolerance is difficult to estimate. It has recently been reported that myopathy is increased by <0.1% in individuals on statins than those on placebo (Amarenco et al., 2006; Ridker et al., 2008; Armitage et al., 2009; Newman et al., 2019). Randomized controlled trials using strict criteria to define myopathy suggested that prevalence is 1–3%. In studies with a more inclusive definition of statin intolerance, prevalence could be as high as 10–25% of cases (Oh et al., 2007).

Several genetic variants have been identified to be potentially associated with statin ADRs through genome-wide, exome-wide, and candidate gene studies. However, the impact of these variants on cholesterol reduction on a population level has not been understood (Canestaro et al., 2014; Brunham et al., 2018; Turner and Pirmohamed, 2019; Ward et al., 2019). In the present retrospective observational study, single nucleotide polymorphisms (SNPs) in the genes of ATP-binding cassette transporter B1 (*ABCB1*), Solute Carrier Organic Anion Transporter Family Member 1B1 (*SLCO1B1*), Leukocyte Immunoglobulin Like Receptor B5 (*LILRB5*), and Cytochromes P450 (*CYP*) family having known associations with statin ADRs were selected to assess their statin efficacy using electronic health records.

### ABCB1

Polymorphisms in *ABCB1* play a vital role in the lipid-lowering response of statins. Variants such as rs1128503 (Gly412Gly, 1236C>T), rs2032582 (Ser893Ala, 2677G>A/T), and rs1045642 (Ile1145Ile, 3435C>T) have been linked to statins pharmacokinetics and statin tolerability (Fiegenbaum et al., 2005; Becker et al., 2009; Hoenig et al., 2011). In a study by Fiegenbaum et al. (2005), the 3435T variants at rs1045642 were associated with decreased risk of myalgia for people treated with simvastatin compared to allele C.

In another study, the T allele variants in rs1045642 were more frequently present in patients on atorvastatin who experienced muscle symptoms compared to those without the variant allele (Hoenig et al., 2011).

### LILRB5

Leukocyte immunoglobulin like receptor B5 is highly expressed in skeletal muscle, liver, and gallbladder. *LILRB5* rs12975366 (Asp247Gly, T>C) was associated with important indicators of muscle damage such as serum CK (creatinine phosphokinase) and lactate dehydrogenase (LDH) levels as well as with statin intolerance and statin-induced myalgia. Individuals homozygous for the wild Asp247 (TT) genotype were more likely to experience elevated CK and LDH levels as well as statin intolerance (Dubé et al., 2014; Kristjansson et al., 2016; Siddiqui et al., 2017).

### SLCO1B1

Many studies extensively focused on *SLCO1B1* polymorphisms and their association with statin-induced myopathy (Puccetti et al., 2010; Donnelly et al., 2011; Brunham et al., 2012).

The *SLCO1B1* rs4149056 (Val174Ala, 521T>C) reduces hepatic uptake of statins. Recessive carriers of the variant experience a higher rate of ADRs (Link et al., 2008; Donnelly et al., 2011). *SLCO1B1* rs2306283 (Asp130Asn, 388 A>G) is a gain of function variant and is associated with statin tolerance (Donnelly et al., 2011).

### Cytochrome P450 enzyme: *CYP3A4* and *CYP3A5*

Cytochromes P450 is a superfamily of enzymes involved in the metabolism of several drugs including statins. Variants in *CYP3A4* (rs2740574), *CYP3A5* (rs776746) have been shown to affect statin intolerance (Wilke et al., 2005; Becker et al., 2010).

### Statin Response

Statin response is measured by reduction of cholesterol, typically LDL cholesterol. Recently, research has determined that non-high-density lipoprotein (non-HDL) cholesterol rather than LDL cholesterol is a better predictor of long-term residual cardiovascular risk (CV) risk in statin-treated individuals (Johannesen et al., 2021). Calculating non-HDL concentration provides a simple way to assess the total amount of pro-atherogenic lipoproteins (apolipoprotein B, i.e., *apoB*). Guidelines from the American Heart Association (AHA), European Society of Cardiology (ESC), and European Atherosclerosis Society (EAS) indicate using non-HDL cholesterol (non-HDL-C) calculated as total cholesterol minus HDL cholesterol to estimate the CV risk (Grundy et al., 2019; Mach et al., 2020; Johannesen et al., 2021).

There remains scepticism around ADRs to statin therapy. A recently concluded cross-over trial has found non-specific complaints of intolerance, i.e., equivalent rates of adverse effects reported, while on statins or placebo (Herrett et al., 2021). However, if ADR and indeed the associated genetic variants result in poor compliance or adherence to statin therapy, a knock-on effect would be observed on cholesterol reduction. Here, we examine variants associated with statin ADRs to determine if they impact non-HDL cholesterol response in the 6months following commencement of statin therapy. We hypothesize that these variants would impact statin efficacy by lowering compliance with statin use.

## Materials and Methods

### Study Design

This study utilizes data from two cohorts that are part of the Tayside Bioresource, University of Dundee: The Genetics of Diabetes Audit and Research in Tayside Scotland (GoDARTS) and Scottish Health Research Register and Biobank (SHARE). Both cohorts are based in the Tayside Region of Scotland, United Kingdom. Both cohorts have genetic biobanks alongside linked electronic health records and community prescribing records. All participants in GoDARTS and SHARE provide informed consent for their medical records to be anonymized and linked to biobanks for clinical and epidemiological research. The cohorts have been used extensively for pharmacogenetic research: to establish associations between statin intolerance and genetic variants, such as *SLCO1B1* and *LILRB5* genotypes (Donnelly et al., 2011; Siddiqui et al., 2017). These cohorts were also used in the discovery of the association between variants of the *F5* gene and an increased risk of ADRs to ACE-I therapy (angiotensin-converting enzyme inhibitors; Maroteau et al., 2020).

These cohorts comprise a consented bioresource with longitudinal follow-up containing complete electronic health records from the same local population. Details of the individual cohorts have been described elsewhere (McKinstry et al., 2017; Hébert et al., 2018). For the purposes of the current study, these cohorts were analyzed collectively as they are from the same base population, data are sourced identically and held in the same International Organization for Standardization 27,001 – and Scottish Government accredited secure safe haven. This approach substantially improves the statistical power of this analysis and overcomes the obstacle faced by most pharmacogenetic studies of insufficient power to detect effects.

### Study Population

The study period was from 1st January 1990 to 31st January 2018. Prescribing and clinical data of cohorts were available from 78,534 individuals. The data linkage includes basic demographics, community prescribing records, biochemistry data from the region-wide clinical laboratory, Scottish Morbidity Records (SMR), detailing International Statistical Classification of Diseases and Related Health Problems (ICD) 9 and 10 codes for hospital admissions. The use of electronic linkage allows access to automatically updated NHS data, which includes hospital admissions, laboratory results, and the provision and fulfilment of prescriptions. Together these were used to characterize statin usage patterns, non-HDL cholesterol response, comorbidities such as CV disease, type 2 diabetes.

### Study Definitions

Data for non-HDL-C was sourced from biochemistry files. Sex and age were determined from demographic data. Type 2 diabetes status from the Scottish Care Information – Diabetes Collaboration (Scottish Diabetes Survey Monitoring Group, 2011). Major adverse cardiovascular events (MACE) were determined using hospital admissions data. All prescribing features such as statin type, dose, statin switching, duration of therapy, and adherence were determined using community prescribing data.

### Statin Efficacy Using Non-HDL-C Response to Therapy

Baseline non-HDL-C (pre-treatment value) was calculated as the nearest value available before statin initiation. The first available non-HDL-C measurement available between 28 and 180days after statin initiation was used. The non-HDL-C reduction was calculated as the difference between post-statin and pre-statin non-HDL-C (mmol/L). Absolute reductions are quoted in the text and tables throughout.

### Statins

Individuals who changed statin type before the non-HDL-C measurement were defined as switchers. Duration of statin therapy was defined as the period between the first statin prescription and the follow-up non-HDL-C measure. The duration of therapy was calculated in days and then divided into 28days to reflect the standard pack size of dispensed statin. To account for differences in potency among statin types, we used a simvastatin equivalent daily dose (Maron et al., 2000), and the mean of all doses during the follow-up was used as a covariate in the analysis. Any reduction or increase of the dose was also identified. Dose reduction before the first non-HDL-C reading was used as one of the predictors of statin intolerance. The percentage of daily coverage (PDC) was used as an indicator of adherence to medication, which can also indicate tolerability of statins. To do this, the quantity of dispensed pills (using pack size information) was calculated. Then the number of days of coverage was calculated based on dates of the first and last prescribed statins. Finally, using prescribing directions (e.g., 1/day or 2/day), we determined if the number of pills dispensed was sufficient for coverage over the period of study. The formula used has been described and used previously (Siddiqui et al., 2017).

### Selection of Statin ADR Variants

Seven SNPs from five different genes were identified through recent systematic reviews (Canestaro et al., 2014; Turner and Pirmohamed, 2019; Ward et al., 2019; Kee et al., 2020) and were selected based on their association with simvastatin and atorvastatin ADRs.

In order to detect genotyping errors, all SNPs were tested for the Hardy-Weinberg equilibrium. We considered the following variants: *ABCB1* rs1128503, *ABCB1* rs1045642, *SLCO1B1* rs4149056 and rs2306283, *LILRB5* rs12975366, *CYP3A4* rs2740574, and *CYP3A5* rs776746.

*Post hoc*, on the basis of the variant effects (dominant, recessive, etc.) and their association with non-HDL-C response to statins, we developed a two-SNP unweighted risk score by considering risk alleles from both *ABCB1* rs1045642 and *LILRB5* rs12975366. There are two levels of this risk score; the protective genotypes were grouped into level 0 (individuals with *LILRB5* rs12975366 genotypes CC or TC and *ABCB1* rs1045642 genotype CC were classified as protected), while individuals with risky genotypes were grouped into level 1 (*LILRB5* rs12975366 genotype TT+ABCB1 rs1045642 genotypes CT or TT) and were classified as at risk of poor response to statins.

### Statistical Methods

Continuous data were presented as a mean and SD; categorical data were expressed as counts and proportions. Analyses were carried out in the entire study population and then was restricted to simvastatin and atorvastatin users only. The association of non-genetic covariates with the outcome of non-HDL-C response was examined using univariate linear regression. Next, the univariate effect of the genetic variants with non-HDL-C response was examined in additive, recessive, and dominant models to determine the genetic effect model based on value of *p* and in concordance with literature. Subsequently, the appropriate genetic effect was examined in models adjusted for features of statin intolerance and in a model adjusted for all measured potential confounders. In the first adjusted model, features of statin intolerance were adherence to therapy (PDC was used as surrogate), switching to another type of statins, and dose reduction. In the second multivariable model, covariates added were the average dose of statin, duration of therapy, the diabetic status of the participant, a history of MACE, and finally, the model was adjusted for baseline level non-HDL-C. Analyses were conducted for each variant, with the hypothesis that they would be associated with statin response. However, these associations are likely to be confounded by statin intolerance and other measured confounders. Therefore, we selected variants that were significant after adjustment for all measured confounders. This included testing for epistasis and non-additive effects. Given the *a priori* hypothesis, results for SNP-wise association testing were considered statistically significant at a 5% level of significance. However, a correction for multiple testing (seven SNPs, three genetic models resulting in 21 independent test) was applied for the two-SNP risk score and results in a threshold of value of *p*<0.002 for significance.

Guidelines and Guidance STrengthening the REporting of Genetic Association Studies (STREGA) were used to report this study (Little et al., 2009). All Statistical analysis was performed with SAS statistical software version 9.4 (SAS Institutes, Cary, NC, United States).

## Results

A total of 9,401 statin users with genotypic information met study inclusion criteria. A population flow chart details the definition of the study population and reasons for exclusion (Supplementary Figure 1). Briefly, of a total of 37,990 statin users, only 19,280 had the necessary baseline and follow-up non-HDL-C measured, of which 9,401 had genotype data available.

### Demographics and Clinical Characteristics

At the time of commencement of statin therapy, the mean age of the participants was 63years (SD±10.97). Females in the cohort constituted 45.3% of the total population (Table 1). About 71.4% of participants had type 2 diabetics and 18.6% had a history of prevalent CV disease before starting statin therapy. The majority of participants were initiated on simvastatin (74.7%) or atorvastatin (19.4%) therapy, of which 3.1% switched therapy to another type of statin. About 38.6% of cases were prescribed a starting dose of 20mg of simvastatin or an equivalent dose of other statins.

**Table 1:** Demographic and clinical descriptions of the study population.

Variables	First measurement within 6months post-statin therapy (*n*=9,401)	Simvastatin and atorvastatin users (*n*=8,843)
Age at starting therapy, mean (SD)	63.06 (10.97)	63.03 (11.01)
Sex		
Female, *n* (%)	4,262 (45.3)	4,023 (45.5)
BMI kg/m^2^, (*n*=8,107), mean (SD)	30.49 (6.06)	30.49 (6.08)
Pre-statin non-HDL-C, mmol/L mean (SD)	4.43 (1.19)	4.42 (1.19)
Post-statin non-HDL-C, mmol/L mean (SD)	2.98 (1.03)	2.94 (1.01)
Mean absolute reduction in non-HDL-C, mmol/L (SD)	1.45 (1.04)	1.47 (1.04)
Median percentage reduction of non-HDL-C, % (IQR)	35.7 (21.1–45.5)	36.5 (22.4–45.9)
The first statin prescribed		
Simvastatin, *n* (%)	7,020 (74.7)	7,020 (79.4)
Atorvastatin, *n* (%)	1,823 (19.4)	1,823 (20.6)
Starting Simvastatin equivalent dose, mg (%)	20 (38.6)	20 (39.8)
Statin switchers before the measurement, *n* (%)	294 (3.1)	268 (3)
Mean duration of statin therapy/28days, periods (SD)	2.96 (1.43)	2.96 (1.43)
Records of dose change before measurement		
Dose reduction	4,589	4,224
Dose increase	5,868	5,393
Mean adherence (SD)	1.54 (0.79)	1.54 (0.78)
Type 2 diabetes mellitus		
Yes, *n* (%)	6,715 (71.4)	6,285 (71.1)
History of MACE		
Prior to statin therapy, *n* (%)	1,749 (18.6)	1,603 (18.1)

### Statin Mediated Non-HDL-C Response

Pre-treatment non-HDL-C levels were measured at a median of 12days (IQR: 4–35days) before statin initiation. Post-treatment non-HDL-C measures were taken at a median of 75days (IQR: 49–112days) after commencing therapy. The mean baseline non-HDL-C level was 4.43 (±1.19) mmol/L, and the mean on-treatment change of non-HDL-C levels was calculated as an absolute reduction of 1.45 (±1.0) mmol/L. The difference in non-HDL-C levels was also calculated as percentage change from pre-treatment, where the median percentage reduction was 35.7% (IQR=21.1–45.5%; Table 1).

### Non-genetic Predictors of Non-HDL-C Response to Statins

Multiple covariates were significantly associated with non-HDL-C response to statin therapy; baseline non-HDL-C level was the major predictor of non-HDL-C reduction within 6months of commencing statin therapy (beta 0.53 CI: 0.51, 0.54; *p*<0.001). PDC, a surrogate for adherence to therapy, was also a significant predictor of non-HDL-C reduction (beta 0.26 CI: 0.23, 0.28; *p*<0.001). The significant results of univariate regression of non-genetic variables and non-HDL cholesterol response are presented in Supplementary Table 1.

### Association of Statin ADR Variants With Non-HDL-C Cholesterol Response to Statins

Minor allele frequencies of the variants were found to be similar to a reference white European population (Karczewski et al., 2020; Supplementary Table 2). The allele frequencies were in Hardy-Weinberg equilibrium for all seven SNPs.

We analyzed the effect of the variants on non-HDL-C in recessive, dominant, and additive genetic models, and the appropriate model was selected for further analyses (Supplementary Table 3). We examined the association of all the ADR variants with statin response in models adjusted for all confounders (Table 2). The only variants associated with statin response were in *ABCB1* rs1045642 (Ile1145Ile, 3435C>T; Table 3) and *LILRB5* rs12975366 (Asp247Gly, T>C; Table 4). Other selected variants did not show any significant association with change in non-HDL-C response in main effects or adjusted models (Supplementary Tables 4–9).

**Table 2:** Univariate effects of statin ADR variants on non-HDL-C reduction.

Gene/SNP	Genetic effect	Statin specificity	*p* (adjusted model)^*^
ABCB1/rs1128503	Dominant	Simvastatin/Atorvastatin	0.278
ABCB1/rs1045642	Recessive	Simvastatin/Atorvastatin	0.017
SLCO1B1/rs4149056	Recessive	Simvastatin/Atorvastatin	0.56
SLCO1B1/rs2306283	Dominant	Simvastatin/Atorvastatin	0.380
LILRB5/rs12975366	Dominant	Not specific	0.03
CYP3A4/rs2740574	Recessive	Simvastatin/Atorvastatin	0.140
CYP3A5/rs776746	Dominant	Simvastatin/Atorvastatin	0.534

* Model adjusted for all measured confounders.

**Table 3:** Effect of ABCB1 (rs1045642686 3435C>T) on the absolute reduction in non-HDL-cholesterol in simvastatin and atorvastatin users.

Variables	Effect estimate (95% CI)		
	Univariate analysis (Model 1)	Model 2	Model 3
*ABCB1* rs1045642	0.09(0.04, 0.14)^**^	0.08(0.03, 0.13)^**^	0.05(0.01, 0.1)^*^
Percentage of daily coverage	-	0.27(0.25, 0.30) ^**^	0.22(0.19, 0.24)^**^
Switching	-	−0.21(−0.35, −0.08)^**^	−0.21(−0.33, −0.09)^**^
Dose reduction	-	−0.25(−0.36, −0.13)^**^	−0.18(−0.27, −0.08)^**^
Mean dose	-	-	0.006(0.005, 0.007)^**^
Duration of statin therapy	-	-	−0.04(−0.06, −0.03)^**^
Type 2 diabetes	-	-	−0.13(−0.17, −0.09)^**^
History of mace	-	-	−0.04(−0.09, 0.01)
Non-HDL-C (baseline)	-	-	0.46(0.45, 0.48)^**^

Model 1: univariate effect, Model 2: features of statin intolerance, and Model 3: features of statin intolerance and important comorbidities.

* *p*<0.05;

** *p*<0.005.

**Table 4:** Effect of LILRB5 (rs12975366) with the absolute reduction in non-HDL cholesterol to all statin treatment.

Variables	Effect estimate (95% CI)		
	Univariate analysis (Model 1)	Model 2	Model 3
*LILRB5* rs12975366	0.04(−0.01,0.08)	0.04(−0.01,0.09)	0.05(0.01,0.08)^*^
Percentage of daily coverage		0.27(0.24,0.30)^**^	0.21(0.19,0.24)^**^
Switching		−0.31(−0.43,−0.19)^**^	−0.25(−0.36,−0.14)^**^
Dose reduction		−0.08(−0.12,−0.04)^**^	−0.18(−0.22,−0.14)^**^
Mean dose		-	0.006(0.005,0.007)^**^
Duration of statin therapy		-	−0.04(−0.06,−0.03)^**^
Type 2 diabetes		-	−0.12(−0.16,−0.08)^**^
History of mace		-	−0.04(−0.09,0.01)
Non-HDL-C (baseline)		-	0.47(0.45,0.49)^**^

Model 1: univariate effect, Model 2: features of statin intolerance, and Model 3: features of statin intolerance and important comorbidities.

* *p* <0.05;

** *p*<0.005.

### *ABCB1* and *LILRB5* Effects

We found that the *ABCB1* rs1045642 (Ile1145Ile, 3435C>T) genotype as a recessive trait was associated with a significant reduction in non-HDL-cholesterol levels (beta 0.09 CI: 0.04, 0.14; *p*=0.001). In models adjusted for features of statin usage, baseline non-HDL-C, type 2 diabetes, CVD, the outcome estimates were still significant. Individuals homozygous for the minor (C) allele had 0.08mmol/L greater reduction of non-HDL-C (CI: 0.03, 0.13; *p*=0.003) compared to carriers of the (T) allele (Table 3). The effect of the *LILRB5* rs12975366 variant was found to be dominant. In an adjusted model, carriers of (C) allele at rs12975366 had a significantly greater reduction of non-HDL-C (beta 0.04 CI: 0.004, 0.08; *p*=0.03) compared to non-carriers (Table 4).

**Figure 1:**
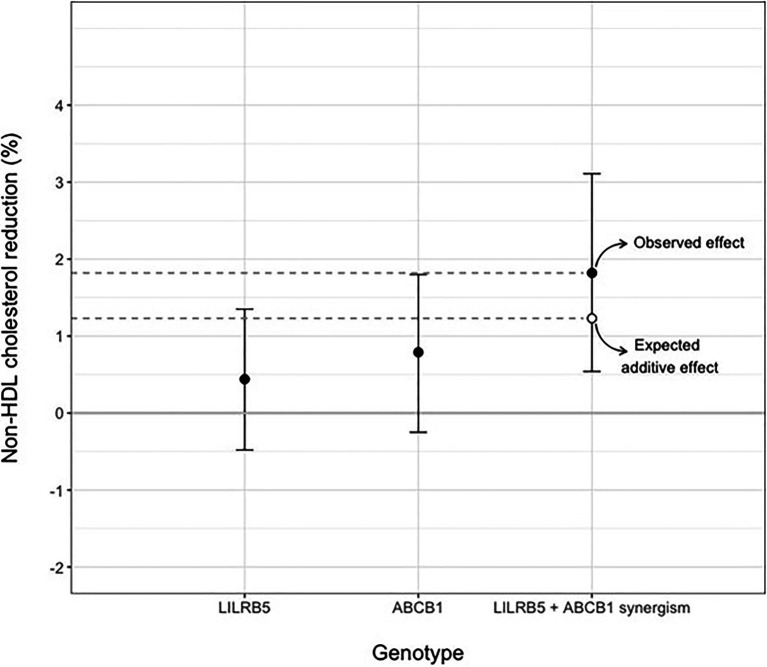
Synergistic effect of LILRB5 and ABCB1 two-variant risk score on percent reduction of non-HDL cholesterol in simvastatin and atorvastatin users. The observed effect was a reduction of 1.82% whereas the expected effect was 1.23%.

We tested the interaction between variants in *ABCB1* and *LILRB5* in a model also adjusted for the main effect of these variants. The interaction term was found to be significant (*p*=0.001). The most significant effect was observed in carriers of both variants (beta 0.14, CI: 0.08, 0.21; *p*<0.001) compared to non-carriers. Based on the significant interaction, we developed a two-variant risk score by combining the recessive *ABCB1* and dominant *LILRB5* variants. Carriers of both *ABCB1* (CC) variant and the protective variants for *LILRB5* (C allele) carriers had 0.1mmol/L (CI: 0.05, 0.16; *p*<0.001) reduction in non-HDL-C compared to non-carriers of the *ABCB1* and *LILRB5* variants (Supplementary Table 10). The combined effect of the *ABCB1* rs1045642 and the *LILRB5* rs12975366 variants was 1.61% of non-HDL-C reduction. In comparison, the expected additive effect would be 0.95% (Table 5 and Figure 1), suggesting that the genetic effects are synergistic. Since *ABCB1* is involved in the pharmacokinetics of simvastatin and atorvastatin only, we restricted our analyses to individuals prescribed those two statins. We found that the main effect of the two-SNP risk score was strongest in subjects prescribed simvastatin (beta 0.16, *p*<0.001, *n*=6,411; Supplementary Table 11) and slightly weaker in those prescribed either simvastatin or atorvastatin (beta 0.14, *p*<0.001, *n*=8,070; Table 6). In this sub-group, the two-SNP risk score in an adjusted model improved non-HDL-C response by 1.82%, whereas the expected additive effect would be 1.23% (Table 5), confirming the synergistic nature of the interaction in adjusted and statin-specific models.

**Table 5:** Two-variant risk score for percentage reduction in non-HDL cholesterol.

	Statin type	Effect estimate (95% CI)		
		*LILRB5* rs12975366 *n* =8569	*ABCB1* rs1045642 *n* =9256	Two-SNP risk score *n* =8569
Percentage reduction of non-HDL-C in adjusted models	All statins	0.45(−0.45,1.35)	0.5(−0.5,1.5)	1.61(0.35,2.87)^**^
	Simvastatin+atorvastatin	0.44(−0.48,1.35) *n* =8070	0.79(−0.25,1.8) *n* =8709	1.82(0.54,3.11)^**^

Models shown were adjusted for all features of statin intolerance, sex, age, BMI, daily dose, duration of therapy, switching therapy, prevalent type 2 diabetes, history of MACE, and baseline non-HDL cholesterol.

** *p*<0.005.

**Table 6:** Effect of LILRB5 and ABCB1 two-variant risk score on the absolute reduction of non-HDL cholesterol in simvastatin and atorvastatin users (n =8,070).

Variable	Effect estimate (95% CI)		
	Univariate analysis (Model 1)	Model 2	Model 3
*LILRB5* rs12975366 (CC or TC)+*ABCB1* rs1045642 (CC) vs. *LILRB5* rs12975366 (TT)+*ABCB1* rs1045642 (CT or TT)	0.14(0.08,0.21)^**^	0.13(0.07,0.19)^**^	0.10(0.04,0.15)^**^
Percentage daily coverage	-	0.27(0.24,0.30)^**^	0.22(0.19,0.24)^**^
Switching	-	−0.31(−0.44,−0.18)^**^	−0.24(−0.35,−0.13)^**^
Dose reduction	-	−0.06(−0.11,−0.02)^*^	−0.15(−0.19,−0.12)^**^
Mean dose	-	-	0.006(0.005,0.007)^**^
Duration of statin therapy	-	-	−0.04(−0.06,−0.03)^**^
Type 2 Diabetes	-	-	−0.12(−0.17,−0.08)^**^
History of MACE	-	-	−0.04(−0.09,0.01)
Non-HDL-Cat baseline	-	-	0.48 (0.46,0.49)^**^

Model 1: univariate effect, Model 2: features of statin intolerance, and Model 3: features of statin intolerance and important comorbidities.

* *p*<0.05;

** *p*<0.005.

## Discussion

This study, leveraging detailed genetic, clinical, and drug dispensing data from nearly 9,000 statin users, finds that two statin ADR variants in *ABCB1* and *LILRB5* are associated synergistically with non-HDL-cholesterol response to statin therapy. Together, individuals homozygous for the C allele in rs1045642 *ABCB1* and carriers of the C allele in rs12975366 *LILRB5* were associated with 0.14mmol/L greater reduction of non-HDL-C in response to simvastatin or atorvastatin therapy compared to those carriers of both the T allele in rs1045642 and those homozygous for the T allele in rs12975366. In main effects analyses, the actual observed effect was greater than the expected additive effect of these two variants. This effect was more pronounced when considering the percentage reduction of non-HDL-C as opposed to the absolute difference. The expected additive effect would be 1.23%, whereas the observed effect was a 1.82% better reduction in variant carriers. Crucially, there was no significant association between these variants and baseline non-HDL-cholesterol or the duration of statin therapy.

Although, some previous studies have found a higher post-treatment reduction of LDL-C in individuals carriers of the T variant genotype at rs1045642 (Kajinami et al., 2004; Kadam et al., 2016), results were inconclusive and a metanalysis indicated that CC variant was associated with decreases in LDL-C levels upon statin treatment when compared to the TT variation (Su et al., 2015). We report that individuals with the homozygous CC variant had 0.09mmol/L higher reduction of non-HDL-C in comparison to those carriers of the T allele.

*LILRB5* rs12975366 did not significantly predict the absolute non-HDL-C reduction univariately, but controlling for confounders and crucial covariates including baseline non-HDL-C in multiple regression models allowed us to estimate a less biased association between the Asp247Gly variant and the absolute reduction of non-HDL-C level. The genotype significantly predicted the percentage reduction of non-HDL-C in both univariate and adjusted models. We hypothesize that together carriers of the C allele of rs12975366 in *LILRB5*, which has been shown to increase statin tolerance, and the CC genotype of rs1045642 in *ABCB1*, which impairs statin excretion from the liver leading to a higher hepatic concentration, result in an enhanced response to the drug.

A limitation of the study is that over 94% of the population were simvastatin or atorvastatin users. Therefore, the results can only be generalizable to populations prescribed either of these drugs. Since these two statins share pharmacokinetic pathways, particularly since they are both substrates for the hepatic efflux transporter *ABCB1*, the results are likely to apply to users of either statin. However, the effects observed for the *LILRB5* variant are not specific to the type of statin as the original effects of the variant were observed in users of simvastatin, atorvastatin, and rosuvastatin and since this is not a pharmacokinetic variant. Further analyses in large observational cohorts are necessary to understand the relationship of statin ADR variants with other statins such as rosuvastatin and pravastatin.

Furthermore, these results would need to be replicated in *post hoc* analyses of randomized clinical trials and in pharmacokinetic studies in order to assess the value of clinical implementation.

Additionally, due to insufficient high-quality genetic data, a polymorphic variant in *ABCB1* (rs2032582) was not examined in this study. This variant forms a haplotype along with the two other *ABCB1* variants examined in this study. However, as documented, the haplotype effect is largely driven by the variant, we have examined, rs1045642.

The lack of association with *SLCO1B1* is surprising as it is the best-documented statin ADR variant. A *SLCO1B1* risk score was also created based on the described haplotype effect by Donnelly et al. (2011), who also did not find the genetic risk score to be associated with LDL-c response in adjusted models. This gene risk score was also not associated with differential response to statins. Similar to our findings, no significant differences in lipid-lowering effect between different *SLCO1B1* genotypes were reported in different studies including genome-wide association studies conducted in white Europeans (Turner and Pirmohamed, 2019; Chen et al., 2020). In a meta-analysis of 13 studies of the association between *SLCO1B1* polymorphisms and the effectiveness of statin in lipid reduction, it was concluded that both 521C and 388G do not affect the lipid-lowering effects of statins. However, in two different sub-analysis one for subjects on a long term treatment of statins (>6months), and another for individuals of non-Asian ethnicities, results showed that those with the wild variant TT had a significant more LDL reduction compared with CC and TC variants (Dai et al., 2015). Similarly, no significant association between haplotype and mean percentage reduction in lipid and lipoprotein levels after simvastatin treatment for 6months was reported in a study by Sortica et al. (2012).

A potential explanation for this lack of association is that the total hepatic exposure to a statin may not be significantly decreased by the change of hepatic uptake in the carriers of the alternative allele and that the effect is more significant on plasma exposure. Therefore, carriers of the minor allele have an increased risk of ADR without a remarkable change in efficacy. Hence, the association between the *SLCO1B1* genotype and ADR is more consistent than its association with the cholesterol-lowering effect of statins. It is also possible that hepatic concentration of statin and statin metabolites for *SLCO1B1* variant carriers is enough to show a lipid-lowering effect at higher daily doses and that the effect of the genetic variant may only appear at lower daily doses. Donnelly et al. (2011) reported a significant association of rs4149056 (Val174Ala) with a higher incidence of statin intolerance and lower LDL-C response. However, when adjusted for features of statin intolerance, the effect was non-significant. Further, once statin-intolerant individuals were removed from the analysis, the association between *SLCO1B1* genotypes and LDL-C response remained non-significant. This result highlights the possibility that variants in this gene have a non-pleiotropic effect on statin ADRs (Donnelly et al., 2011).

A *post hoc* power analysis shows that the study is sufficiently powered to detect non-HDL-C changes as small as 0.07mmol/L for genetic variants with MAF greater than 0.42. Whereas, for variants such as rs4149056 (Val174Ala; MAF=0.16) the minimum detectable difference would be 0.2mmol/L. Therefore, it is possible that this study is insufficiently powered to detect effects for rs4149056 (Val174Ala) variant in *SLCO1B1* or for rs2740574 in *CYP3A4*.

It is also likely that individuals who were prescribed low doses of statins do not have a high non-HDL-cholesterol lowering requirement. While, we have adjusted for dose, history of MACEs, and baseline non-HDL-C, there may still be residual confounding diluting the genetic effects we report. In our data, the median simvastatin equivalent daily dose was 20mg, and only 5% of patients started on a therapeutic dose less than 10mg daily, which implies that our analysis lacks the statistical power to detect differences in these groups.

The study demonstrates real-world prescribing, behaviors, and effects. The duration of follow-up allows us to avoid heterogeneous effects associated with differential lengths of statin use. With longer follow-up, other confounding factors arise – changes to, e.g., diet, exercise, changes to statin type, and dosing regimens. Some of these are hard to measure. It also reflects the first clinical interaction after the commencement of statin use, where a medical professional assesses the observed efficacy of the statin. This time point is crucial as 66% of the population in our cohort is assessed by the end of these 6months.

## Conclusion

These results highlight the value in genotyping statin ADR variants, as they affect tolerance to statins and statin efficacy. Even though, some of these variants have proven evidence of association with statin ADRs (e.g., variants in SLCO1B1), genetic testing is still limited. Li et al. (2014) compared a group of genotyped patients to a non-genotyped group. They found a significantly greater reduction in LDL-C within the genotyped group compared to non-genotyped. The same group also had more new statin prescriptions as well as better adherence. Interestingly in this study both carriers and non-carriers of the risk alleles benefited from genetic testing, which may suggest that genotyping may even provide benefits to the patient regardless of the test result.

Our two-SNP risk score was associated with a 1.82% change in statin treated individuals. Oni-Orisan et al. (2018) recently demonstrated that doubling of statin dose was associated with an approximately 5–10% reduction in non-HDL cholesterol. Thus, our observed reduction due to the two-SNP risk score is equivalent to a 36–73% increase in statin dose. With the polemics around the nocebo effect in statin-treated individuals (Herrett et al., 2021), such findings carry weight as they demonstrate an effect on statin efficacy independent of poor adherence.

## Data Availability Statement

The data analyzed in this study is subject to the following licenses/restrictions: Restrictions applied to datasets. The datasets presented in this article are not readily available as they contain individual-level identifiable information. All analyses of anonymized data are performed in an International Organization for Standardization 27,001– and Scottish Government–accredited secure safe haven. Data requests can be initiated by contacting the corresponding author. Requests to access these datasets should be directed to MS (m.k.siddiqui@dundee.ac.uk).

## Ethics Statement

The GoDARTS studies involving human participants were reviewed and approved by Tayside Medical Ethics Committee 053/04 and East of Scotland Ethics committee NHS REC 13/ES/0020. The patients/participants provided their written informed consent to participate in this study.

## Author Contributions

AM, MC, MB, CP, and MS contributed to the conception and design of the study. AM and MC performed the data cleaning and statistical analysis. MB assisted with statistical analyses and interpretation. CM, AT, AD, RP, AT, and CP assisted with data curation, interpretation, and critical revision of the manuscript. AM and MS wrote the first draft of the manuscript and critically revised the manuscript. All authors contributed to the article and approved the submitted version.

## Funding

GoDARTS was funded and supported by the Wellcome Trust, Tenovus Scotland, and Diabetes UK grants. SHARE is NHS Scotland Research (NRS) infrastructure initiative and it was funded by the Chief Scientists Office of the Scottish Government. Additional Funding and initiation of the spare blood retention at NHS Tayside was supported by the Wellcome Trust Biomedical Resource (award number 099177/Z/12/Z).

## Conflict of Interest

The authors declare that the research was conducted in the absence of any commercial or financial relationships that could be construed as a potential conflict of interest.

The reviewer YZ declared a past co-authorship with the authors AD, CP, and MS to the handling editor.

## Publisher’s Note

All claims expressed in this article are solely those of the authors and do not necessarily represent those of their affiliated organizations, or those of the publisher, the editors and the reviewers. Any product that may be evaluated in this article, or claim that may be made by its manufacturer, is not guaranteed or endorsed by the publisher.

## References

[ref1] Alfirevic A. Neely D. Armitage J. Chinoy H. Cooper R. G. Laaksonen R.. (2014). Phenotype standardisation for statin-induced myotoxicity. Clin. Pharmacol. Ther. 96, 470–476. doi: 10.1038/clpt.2014.121, PMID: 24897241 PMC4172546

[ref2] Amarenco P. Bogousslavsky J. Callahan A. 3rd Goldstein L. B. Hennerici M. Rudolph A. E.. (2006). High-dose atorvastatin after stroke or transient ischemic attack. N. Engl. J. Med. 355, 549–559. doi: 10.1056/NEJMoa061894, PMID: 16899775

[ref3] Armitage J. Bowman L. Collins R. Parish S. Tobert J. (2009). Effects of simvastatin 40 mg daily on muscle and liver adverse effects in a 5-year randomised placebo-controlled trial in 20,536 high-risk people. BMC Clin. Pharmacol. 9:6. doi: 10.1186/1472-6904-9-6, PMID: 19442259 PMC2676245

[ref4] Armitage J. Bowman L. Wallendszus K. Bulbulia R. Rahimi K. Haynes R.. (2010). Intensive lowering of LDL cholesterol with 80 mg versus 20 mg simvastatin daily in 12,064 survivors of myocardial infarction: a double-blind randomised trial. Lancet 376, 1658–1669. doi: 10.1016/S0140-6736(10)60310-8, PMID: 21067805 PMC2988223

[ref5] Baigent C. Keech A. Kearney P. M. Blackwell L. Buck G. Pollicino C.. (2005). Efficacy and safety of cholesterol-lowering treatment: prospective meta-analysis of data from 90,056 participants in 14 randomised trials of statins. Lancet 366, 1267–1278. doi: 10.1016/S0140-6736(05)67394-1, PMID: 16214597

[ref6] Bair T. L. May H. T. Knowlton K. U. Anderson J. L. Lappe D. L. Muhlestein J. B. (2020). Predictors of statin intolerance in patients with a new diagnosis of atherosclerotic cardiovascular disease within a large integrated health care institution: the IMPRES study. J. Cardiovasc. Pharmacol. 75, 426–431. doi: 10.1097/FJC.0000000000000808, PMID: 32079856

[ref7] Becker M. L. Visser L. E. van Schaik R. H. N. Hofman A. Uitterlinden A. G. Stricker B. H. (2009). Common genetic variation in the ABCB1 gene is associated with the cholesterol-lowering effect of simvastatin in males. Pharmacogenomics 10, 1743–1751. doi: 10.2217/pgs.09.105, PMID: 19891551

[ref8] Becker M. L. Visser L. E. van Schaik R. H. Hofman A. Uitterlinden A. G. Stricker B. H. (2010). Influence of genetic variation in CYP3A4 and ABCB1 on dose decrease or switching during simvastatin and atorvastatin therapy. Pharmacoepidemiol. Drug Saf. 19, 75–81. doi: 10.1002/pds.1866, PMID: 19802823

[ref9] Brunham L. R. Baker S. Mammen A. Mancini G. B. J. Rosenson R. S. (2018). Role of genetics in the prediction of statin-associated muscle symptoms and optimisation of statin use and adherence. Cardiovasc. Res. 114, 1073–1081. doi: 10.1093/cvr/cvy119, PMID: 29878063 PMC6014181

[ref10] Brunham L. R. Lansberg P. J. Zhang L. Miao F. Carter C. Hovingh G. K.. (2012). Differential effect of the rs4149056 variant in SLCO1B1 on myopathy associated with simvastatin and atorvastatin. Pharmacogenomics J. 12, 233–237. doi: 10.1038/tpj.2010.92, PMID: 21243006

[ref11] Canestaro W. J. Austin M. A. Thummel K. E. (2014). Genetic factors affecting statin concentrations and subsequent myopathy: a HuGENet systematic review. Genet. Med. 16, 810–819. doi: 10.1038/gim.2014.41, PMID: 24810685 PMC4676271

[ref12] Chen W. J. Wen Y. C. Fox K. M. Shen L. J. Lin L. Y. Qian Y.. (2020). Treatment patterns of lipid-lowering therapies and possible statin intolerance among statin users with clinical atherosclerotic cardiovascular disease (ASCVD) or diabetes mellitus (DM) in Taiwan. J. Eval. Clin. Pract. 26, 1171–1180. doi: 10.1111/jep.13286, PMID: 31646715

[ref13] Dai R. Feng J. Yang Y. Deng C. Tang X. Zhao Y.. (2015). Association between SLCO1B1 521 T > C and 388 A > G polymorphisms and statins effectiveness: a meta-analysis. J. Atheroscler. Thromb. 22, 796–815. doi: 10.5551/jat.26856, PMID: 25832498

[ref14] Donnelly L. A. Doney A. S. Tavendale R. Lang C. C. Pearson E. R. Colhoun H. M.. (2011). Common nonsynonymous substitutions in SLCO1B1 predispose to statin intolerance in routinely treated individuals with type 2 diabetes: a go-DARTS study. Clin. Pharmacol. Ther. 89, 210–216. doi: 10.1038/clpt.2010.255, PMID: 21178985 PMC3353487

[ref15] Dubé M. P. Zetler R. Barhdadi A. Brown A. M. Mongrain I. Normand V.. (2014). CKM and LILRB5 are associated with serum levels of creatine kinase. Circ. Cardiovasc. Genet. 7, 880–886. doi: 10.1161/CIRCGENETICS.113.000395, PMID: 25214527

[ref16] Fiegenbaum M. da Silveira F. R. Van der Sand C. R. Van der Sand L. C. Ferreira M. E. Pires R. C.. (2005). The role of common variants of ABCB1, CYP3A4, and CYP3A5 genes in lipid-lowering efficacy and safety of simvastatin treatment. Clin. Pharmacol. Ther. 78, 551–558. doi: 10.1016/j.clpt.2005.08.003, PMID: 16321621

[ref17] Grundy S. M. Stone N. J. Bailey A. L. Beam C. Birtcher K. K. Blumenthal R. S.. (2019). 2018 AHA/ACC/AACVPR/AAPA/ABC/ACPM/ADA/AGS/APhA/ASPC/NLA/PCNA guideline on the management of blood cholesterol: a report of the American College of Cardiology/American Heart Association task force on clinical practice guidelines. Circulation 139, e1082–e1143. doi: 10.1161/CIR.0000000000000625, PMID: 30586774 PMC7403606

[ref18] Hébert H. L. Shepherd B. Milburn K. Veluchamy A. Meng W. Carr F.. (2018). Cohort profile: genetics of diabetes audit and research in Tayside Scotland (GoDARTS). Int. J. Epidemiol. 47, 380–381. doi: 10.1093/ije/dyx140, PMID: 29025058 PMC5913637

[ref19] Herrett E. Williamson E. Brack K. Beaumont D. Perkins A. Thayne A.. (2021). Statin treatment and muscle symptoms: series of randomised, placebo controlled n-of-1 trials. BMJ 372:n135. doi: 10.1136/bmj.n135, PMID: 33627334 PMC7903384

[ref20] Hoenig M. R. Walker P. J. Gurnsey C. Beadle K. Johnson L. (2011). The C3435T polymorphism in ABCB1 influences atorvastatin efficacy and muscle symptoms in a high-risk vascular cohort. J. Clin. Lipidol. 5, 91–96. doi: 10.1016/j.jacl.2011.01.001, PMID: 21392722

[ref21] Johannesen C. D. L. Mortensen M. B. Langsted A. Nordestgaard B. G. (2021). Apolipoprotein B and non-HDL cholesterol better reflect residual risk than LDL cholesterol in statin-treated patients. J. Am. Coll. Cardiol. 77, 1439–1450. doi: 10.1016/j.jacc.2021.01.027, PMID: 33736827

[ref22] Kadam P. Ashavaid T. F. Ponde C. K. Rajani R. M. (2016). Genetic determinants of lipid-lowering response to atorvastatin therapy in an Indian population. J. Clin. Pharm. Ther. 41, 329–333. doi: 10.1111/jcpt.12369, PMID: 26932749

[ref23] Kajinami K. Brousseau M. E. Ordovas J. M. Schaefer E. J. (2004). Polymorphisms in the multidrug resistance-1 (MDR1) gene influence the response to atorvastatin treatment in a gender-specific manner. Am. J. Cardiol. 93, 1046–1050. doi: 10.1016/j.amjcard.2004.01.014, PMID: 15081455

[ref24] Karczewski K. J. Francioli L. C. Tiao G. Cummings B. B. Alföldi J. Wang Q.. (2020). The mutational constraint spectrum quantified from variation in 141,456 humans. Nature 581, 434–443. doi: 10.1038/s41586-020-2308-7, PMID: 32461654 PMC7334197

[ref25] Kee P. S. Chin P. K. L. Kennedy M. A. Maggo S. D. S. (2020). Pharmacogenetics of statin-induced myotoxicity. Front. Genet. 11:1114. doi: 10.3389/fgene.2020.575678, PMID: 33193687 PMC7596698

[ref26] Kristjansson R. P. Oddsson A. Helgason H. Sveinbjornsson G. Arnadottir G. A. Jensson B. O.. (2016). Common and rare variants associating with serum levels of creatine kinase and lactate dehydrogenase. Nat. Commun. 7:10572. doi: 10.1038/ncomms10572, PMID: 26838040 PMC4742860

[ref27] Li J. H. Joy S. V. Haga S. B. Orlando L. A. Kraus W. E. Ginsburg G. S.. (2014). Genetically guided statin therapy on statin perceptions, adherence, and cholesterol lowering: a pilot implementation study in primary care patients. J. Pers. Med. 4, 147–162. doi: 10.3390/jpm4020147, PMID: 25563221 PMC4263970

[ref28] Link E. Parish S. Armitage J. Bowman L. Heath S. Matsuda F.. (2008). SLCO1B1 variants and statin-induced myopathy–a genome-wide study. N. Engl. J. Med. 359, 789–799. doi: 10.1056/NEJMoa0801936, PMID: 18650507

[ref29] Little J. Higgins J. P. T. Ioannidis J. P. A. Moher D. Gagnon F. von Elm E.. (2009). STrengthening the REporting of Genetic Association Studies (STREGA)—an extension of the STROBE statement. PLoS Med. 6:e1000022. doi: 10.1371/journal.pmed.1000022, PMID: 19192942 PMC2634792

[ref30] Mach F. Baigent C. Catapano A. L. Koskinas K. C. Casula M. Badimon L.. (2020). 2019 ESC/EAS guidelines for the management of dyslipidaemias: lipid modification to reduce cardiovascular risk: the task force for the management of dyslipidaemias of the European Society of Cardiology (ESC) and European Atherosclerosis Society (EAS). Eur. Heart J. 41, 111–188. doi: 10.1093/eurheartj/ehz455, PMID: 31504418

[ref31] Mangravite L. M. Thorn C. F. Krauss R. M. (2006). Clinical implications of pharmacogenomics of statin treatment. Pharmacogenomics J. 6, 360–374. doi: 10.1038/sj.tpj.6500384, PMID: 16550210

[ref32] Maron D. J. Fazio S. Linton M. F. (2000). Current perspectives on statins. Circulation 101, 207–213. doi: 10.1161/01.CIR.101.2.207, PMID: 10637210

[ref33] Maroteau C. Siddiqui M. K. Veluchamy A. Carr F. White M. Cassidy A. J.. (2020). Exome sequencing reveals common and rare variants in F5 associated with ACE inhibitor and angiotensin receptor blocker-induced angioedema. Clin. Pharmacol. Ther. 108, 1195–1202. doi: 10.1002/cpt.1927, PMID: 32496628 PMC10306231

[ref34] McKinstry B. Sullivan F. M. Vasishta S. Armstrong R. Hanley J. Haughney J.. (2017). Cohort profile: the Scottish research register SHARE. A register of people interested in research participation linked to NHS data sets. BMJ Open 7:e013351. doi: 10.1136/bmjopen-2016-013351, PMID: 28148535 PMC5293989

[ref35] Mihaylova B. Emberson J. Blackwell L. Keech A. Simes J. Barnes E. H.. (2012). The effects of lowering LDL cholesterol with statin therapy in people at low risk of vascular disease: meta-analysis of individual data from 27 randomised trials. Lancet 380, 581–590. doi: 10.1016/S0140-6736(12)60367-5, PMID: 22607822 PMC3437972

[ref36] Newman C. B. Preiss D. Tobert J. A. Jacobson T. A. Page R. L. 2nd Goldstein L. B.. (2019). Statin safety and associated adverse events: a scientific statement from the American Heart Association. Arterioscler. Thromb. Vasc. Biol. 39, e38–e81. doi: 10.1161/ATV.0000000000000073, PMID: 30580575

[ref37] Oh J. Ban M. R. Miskie B. A. Pollex R. L. Hegele R. A. (2007). Genetic determinants of statin intolerance. Lipids Health Dis. 6:7. doi: 10.1186/1476-511X-6-7, PMID: 17376224 PMC1832194

[ref38] Oni-Orisan A. Hoffmann T. J. Ranatunga D. Medina M. W. Jorgenson E. Schaefer C.. (2018). Characterization of statin low-density lipoprotein cholesterol dose-response using electronic health records in a large population-based cohort. Circ. Genom. Precis. Med. 11:e002043. doi: 10.1161/CIRCGEN.117.002043, PMID: 30354326 PMC6214660

[ref39] Pedro-Botet J. Climent E. Benaiges D. (2019). Muscle and statins: from toxicity to the nocebo effect. Expert Opin. Drug Saf. 18, 573–579. doi: 10.1080/14740338.2019.1615053, PMID: 31070941

[ref40] Puccetti L. Ciani F. Auteri A. (2010). Genetic involvement in statins induced myopathy. Preliminary data from an observational case-control study. Atherosclerosis 211, 28–29. doi: 10.1016/j.atherosclerosis.2010.02.026, PMID: 20347093

[ref41] Ridker P. M. Danielson E. Fonseca F. A. Genest J. Gotto A. M. Jr. Kastelein J. J.. (2008). Rosuvastatin to prevent vascular events in men and women with elevated C-reactive protein. N. Engl. J. Med. 359, 2195–2207. doi: 10.1056/NEJMoa0807646, PMID: 18997196

[ref42] Schachter M. (2005). Chemical, pharmacokinetic and pharmacodynamic properties of statins: an update. Fundam. Clin. Pharmacol. 19, 117–125. doi: 10.1111/j.1472-8206.2004.00299.x, PMID: 15660968

[ref43] Scottish Diabetes Survey Monitoring Group (2011). Scottish Diabetes Survey. Available at: https://www.diabetesinscotland.org.uk/wp-content/uploads/2019/12/Diabetes-in-Scotland-website-Scottish-Diabetes-Survey-2011.pdf (Accessed May 19, 2021).

[ref44] Selva-O’Callaghan A. Alvarado-Cardenas M. Pinal-Fernández I. Trallero-Araguás E. Milisenda J. C. Martínez M. Á.. (2018). Statin-induced myalgia and myositis: an update on pathogenesis and clinical recommendations. Expert. Rev. Clin. Immunol. 14, 215–224. doi: 10.1080/1744666X.2018.1440206, PMID: 29473763 PMC6019601

[ref45] Siddiqui M. K. Maroteau C. Veluchamy A. Tornio A. Tavendale R. Carr F.. (2017). A common missense variant of LILRB5 is associated with statin intolerance and myalgia. Eur. Heart J. 38, 3569–3575. doi: 10.1093/eurheartj/ehx467, PMID: 29020356 PMC5837247

[ref46] Sortica V. A. Fiegenbaum M. Lima L. O. Van der Sand C. R. Van der Sand L. C. Ferreira M. E.. (2012). SLCO1B1 gene variability influences lipid-lowering efficacy on simvastatin therapy in southern Brazilians. Clin. Chem. Lab. Med. 50, 441–448. doi: 10.1515/cclm.2011.804, PMID: 22505549

[ref47] Su J. Xu H. Yang J. Yu Q. Yang S. Zhang J.. (2015). ABCB1 C3435T polymorphism and the lipid-lowering response in hypercholesterolemic patients on statins: a meta-analysis. Lipids Health Dis. 14:122. doi: 10.1186/s12944-015-0114-2, PMID: 26438079 PMC4594898

[ref48] Taylor B. A. Thompson P. D. (2018). Statin-associated muscle disease: advances in diagnosis and management. Neurotherapeutics 15, 1006–1017. doi: 10.1007/s13311-018-0670-z, PMID: 30251222 PMC6277297

[ref49] Turner R. M. Pirmohamed M. (2019). Statin-related myotoxicity: a comprehensive review of pharmacokinetic, pharmacogenomic and muscle components. J. Clin. Med. 9:22. doi: 10.3390/jcm9010022, PMID: 31861911 PMC7019839

[ref50] Vermes A. Vermes I. (2004). Genetic polymorphisms in cytochrome P450 enzymes: effect on efficacy and tolerability of HMG-CoA reductase inhibitors. Am. J. Cardiovasc. Drugs 4, 247–255. doi: 10.2165/00129784-200404040-00005, PMID: 15285699

[ref51] Ward N. C. Watts G. F. Eckel R. H. (2019). Statin toxicity. Circ. Res. 124, 328–350. doi: 10.1161/CIRCRESAHA.118.312782, PMID: 30653440

[ref52] Wilke R. A. Moore J. H. Burmester J. K. (2005). Relative impact of CYP3A genotype and concomitant medication on the severity of atorvastatin-induced muscle damage. Pharmacogenet. Genomics 15, 415–421. doi: 10.1097/01213011-200506000-00007, PMID: 15900215

