# Supplementary material for: Common Statin Intolerance Variants in ABCB1 and LILRB5 Show Synergistic Effects on Statin Response: An Observational Study Using Electronic Health Records
Source: Front Genet. 2021 Oct 1;12:713181. doi: 10.3389/fgene.2021.713181 (PMC8517257; doi:10.3389/fgene.2021.713181)
Supplement: Supplementary file 1 [file Data_Sheet_1.docx]

# Supplementary materials and methods

Melhem *et al.*


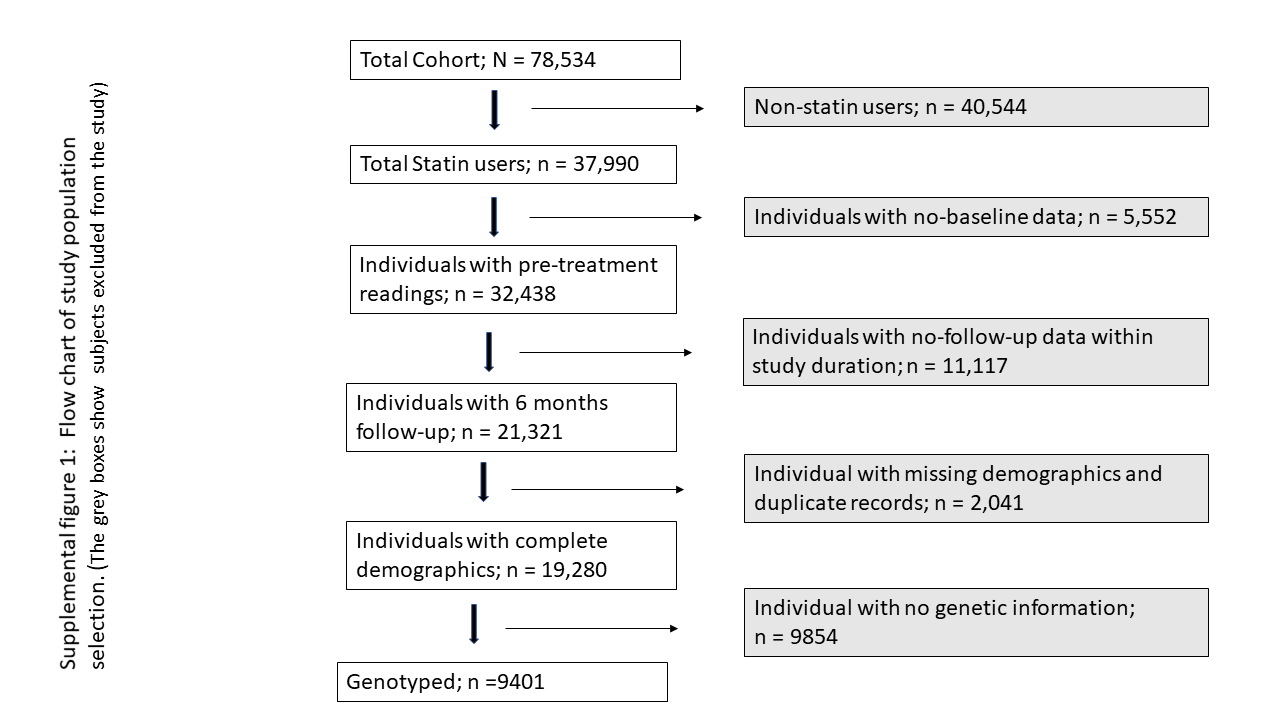


Supplementary Figure 1. Population flow chart. The sample size for each variant analysed differs from the final number (n=9401) based on genetic data quality thresholds applied.

Supplementary table 1. Univariate analysis of demography and main clinical covariables with the absolute non-HDL-C reduction.

| Variable | Effect estimate | P-value | 95% CI | R^2^ |
| --- | --- | --- | --- | --- |
| Sex | 0.14 | <0.001 | 0.10,0.18 | 0.004 |
| Age (years) | 0.003 | 0.01 | 0.001,0.004 | 0.001 |
| BMI KG/M2 | -0.01 | <0.001 | -0.013,-0.005 | 0.003 |
| Type 2 diabetes | -0.26 | <0.001 | -0.31,-0.22 | 0.013 |
| Baseline non-HDL cholesterol (mmol/L) | 0.51 | <0.001 | 0.50,0.53 | 0.342 |
| Switching | -0.25 | <0.001 | -0.37,-0.12 | 0.002 |
| Percentage daily coverage (%) | 0.26 | <0.001 | 0.23,0.28 | 0.038 |
| Mean equivalent dose | 0.002 | <0.001 | 0.001,0.003 | 0.002 |
| Dose reduction | -0.06 | 0.011 | -0.10,-0.01 | 0.001 |
| Duration of statin therapy (/28days) | -0.12 | <0.001 | -0.14,-0.11 | 0.029 |
| Prior history of MACE | -0.14 | <0.001 | -0.19,-0.08 | 0.003 |

*Supplementary Table 2. Details of statin ADR variants*

| Position | SNP | Gene | Amino acid | N | Homozygous major allele count | Heterozygous variant count | Homozygous minor allele count | Minor allele  frequency | Minor allele  frequency in White European reference populations |
| --- | --- | --- | --- | --- | --- | --- | --- | --- | --- |
| chr7:87550285 | rs1128503 | *ABCB1* | Gly412Gly | 9255 | 2897 | 4456 | 1902 | 0.45 (T) | 0.42 |
| chr7:87509329 | rs1045642 | *ABCB1* | Ile1145Ile | 9256 | 2707 | 4525 | 2024 | 0.46 (C) | 0.48 |
| chr12:21178615 | rs4149056 | *SLCO1B1* | Val174Ala | 9367 | 6613 | 2506 | 248 | 0.16 (C) | 0.16 |
| chr12:21176804 | rs2306283 | *SLCO1B1* | Asn130Asp | 9277 | 3620 | 4312 | 1345 | 0.38 (C) | 0.40 |
| chr19:54255498 | rs12975366 | *LILRB5* | Asp247Gly | 8569 | 3195 | 4123 | 1251 | 0.39 (C) | 0.43 |
| chr7:99784473 | rs2740574 | *CYP3A4* | - | 8533 | 8014 | 502 | 17 | 0.03 (C) | 0.03 |
| chr7:99672916 | rs776746 | *CYP3A5* | - | 9284 | 8099 | 1133 | 52 | 0.07 (T) | 0.0 |

Allele frequency of reference populations taken from Haplotype Reference Consortium.

*Supplementary Table 3. SNPs associations with non-HDL-C*

| GENE | SNP | Padditive | Pdominant | Precessive | Padjusted |
| --- | --- | --- | --- | --- | --- |
| *ABCB1* | rs1128503 | 0.332 | 0.175 | 0.878 | 0.278 |
| ***ABCB1*** | **rs1045642** | **0.012** | **0.222** | **0.001** | **0.017** |
| *SLCO1B1* | rs4149056 | 0.689 | 0.949 | 0.264 | 0.56 |
| *SLCO1B1* | rs2306283 | 0.685 | 0.704 | 0.789 | 0.380 |
| ***LILRB5*** | **rs12975366** | **0.151** | **0.124** | **0.500** | **0.03** |
| *CYP3A4* | rs2740574 | 0.785 | 0.962 | 0.201 | 0.140 |
| *CYP3A5* | rs776746 | 0.284 | 0.271 | 0.854 | 0.534 |

*P adjusted is for P-value using the appropriate genetic model, and adjusted for all covariates i.e. demography, features of statin intolerance and important comorbidities.*

Supplementary Table 4. Effect of SLCO1B1 (rs4149056) with the absolute reduction in non-HDL-C in response to simvastatin and atorvastatin treatment (n=8,811)

| Variables | Effect estimate(95%CI) , P-value | | |
| --- | --- | --- | --- |
|  | Univariate analysis  (Model 1) | Model 2 | Model 3 |
| *SLCO1B1* rs4149056 | -0.07(-0.20,0.07), 0.328 | -0.07(-0.20,0.067), 0.329 | -0.03(-0.15,0.08), 0.56 |
| Percentage of daily coverage | - | 0.27(0.24,0.30), <0.001 | 0.21(0.19,0.24), <0.001 |
| Switching | - | -0.33(-0.46,-0.21), <0.001 | -0.28(-0.39,-0.17), <0.001 |
| Dose reduction | - | -0.06(-0.10,-0.01), 0.011 | -0.13(-0.17,-0.09), <0.001 |
| Sex | - | - | -0.01(-0.05,0.03)0.611 |
| Age | - | - | 0.01(0.009,0.013),<0.001 |
| BMI | - | - | -0.004(-0.008,-0.001),0.01 |
| Mean dose | - | - | 0.006(0.005,0.007),<0.001 |
| Duration of statin therapy | - | - | -0.04(-0.06,-0.03),<0.001 |
| Type 2 diabetes | - | - | -0.13(-0.17,-0.09), <0.001 |
| History of mace | - | - | -0.04 (-0.09,0.01), 0.094 |
| Non-HDL cholesterol at baseline | - | - | 0.47(0.45,0.49), <0.001 |

Model 1: univariate effect, Model 2: features of statin intolerance, Model 3: demography ,features of statin intolerance and important comorbidities.

Supplementary Table 5. Effect of SLCO1B1 (rs2306283) with the absolute reduction in non-HDL-C in response to simvastatin and atorvastatin treatment (n=8,762)

| Variable | Effect estimate(95%CI) , P-value | | |
| --- | --- | --- | --- |
|  | Univariate analysis  (Model 1) | Model 2 | Model 3 |
| *SLCO1B1* rs2306283 | -0.02 (-0.06,0.03), 0.477 | -0.02(-0.06,0.03), 0.410 | -0.02(-0.06,0.02), 0.380 |
| Percentage of daily coverage | - | 0.27(0.24,0.30), <0.001 | 0.21(0.18,0.24) , <0.001 |
| Switching | - | -0.32(-0.45,-0.20), <0.001 | -0.28(-0.39,-0.16), <0.001 |
| Dose reduction | - | -0.06(-0.10,-0.01), 0.011 | -0.13(-0.17,-0.10), <0.001 |
| Sex | - | - | -0.01(-0.05,0.03)0.541 |
| Age | - | - | 0.01(0.009,0.013),<0.001 |
| BMI | - | - | -0.004(-0.008,-0.001),0.01 |
| Mean dose | - | - | 0.006(0.005,0.007),<0.001 |
| Duration of statin therapy | - | - | -0.04(-0.06,-0.03), <0.001 |
| Type 2 diabetes | - | - | -0.09(-0.13,-0.04), <0.001 |
| HISTORY OF MACE | - | - | -0.06(-0.11,-0.01), 0.025 |
| Non-HDL cholesterol at baseline | - | - | 0.5(0.48,0.52), <0.001 |

Model 1: univariate effect, Model 2: features of statin intolerance, Model 3: demography ,features of statin intolerance and important comorbidities.

Supplementary Table 6. Effect of ABCB1 (rs1128503) with the absolute reduction in non-HDL-C in response to simvastatin and atorvastatin treatment (n=8,709)

| Variable | Effect estimate(95%CI) , P-value | | |
| --- | --- | --- | --- |
|  | Univariate analysis  (Model 1) | Model 2 | Model 3 |
| *ABCB1* rs1128503 | -0.04(-0.09,0.01), 0.102 | -0.03(-0.08,0.02), 0.206 | -0.02(-0.06,0.02),0.278 |
| Percentage of daily coverage |  | 0.27(0.24,0.30), <0.001 | 0.21(0.18,0.23),<0.001 |
| Switching |  | -0.33(-0.46,-0.21),<0.001 | -0.30(-0.41,-0.19), <0.001 |
| Dose reduction |  | -0.05(-0.10,-0.01) P0.017 | -0.13(-0.17,-0.09), <0.001 |
| Sex | - | - | -0.01(-0.05,0.03)0.561 |
| Age | - | - | 0.01(0.009,0.013),<0.001 |
| BMI | - | - | -0.004(-0.007,-0.001),0.18 |
| Mean dose |  | - | 0.006(0.005,0.007),<0.001 |
| Duration of statin therapy |  | - | -0.04(-0.06,-0.03), <0.001 |
| Type 2 diabetes |  | - | -0.09(-0.14,-0.05), <0.001 |
| History of MACE |  | - | -0.05(-0.1,-0.002),0.042 |
| Non-HDL cholesterol at baseline |  | - | 0.5(0.48,0.52), <0.001 |

Model 1: univariate effect, Model 2: features of statin intolerance, Model 3: demography ,features of statin intolerance and important comorbidities.

Supplementary Table 7. Effect of SLCO1B1 genetic risk score with the absolute reduction in non-HDL-C in response to simvastatin and atorvastatin treatment (n=8,695)

| Variable | Effect estimate(95%CI) , P-value | | |
| --- | --- | --- | --- |
|  | Univariate analysis  (Model 1) | Model 2 | Model 3 |
| *SLCO1B1* genetic risk score | -0.01(-0.05,0.02), 0.507 | -0.02(-0.06,0.02), 0.264 | -0.02(-0.05,0.01), 0.185 |
| Percentage of daily coverage | - | 0.27(0.24,0.30), <0.001 | 0.21(0.18,0.24), <0.001 |
| Switching | - | -0.32(-0.45,-0.19), <0.001 | -0.27(-0.38,-0.16), <0.001 |
| Dose reduction | - | -0.06 (-0.10,-0.14), 0.010 | -0.13(-0.17,-0.09), <0.001 |
| Sex | - | - | -0.01(-0.05,0.03)0.560 |
| Age | - | - | 0.01(0.009,0.013),<0.001 |
| BMI | - | - | -0.004(-0.008,-0.001),0.11 |
| Mean dose | - | - | 0.006(0.005,0.007),<0.001 |
| Duration of statin therapy | - | - | -0.04(-0.06,-0.03), <0.001 |
| Type 2 diabetes | - | - | -0.09(-0.13,-0.04), <0.001 |
| History of mace | - | - | -0.06 (-0.11,-0.01), 0.025 |
| Non-HDL-C at baseline | - | - | 0.5(0.48,0.52), <0.001 |

Model 1: univariate effect, Model 2: features of statin intolerance, Model 3: demography, features of statin intolerance and important comorbidities.

Supplementary Table 8. Effect of CYP3A4 (rs2740574) with the absolute reduction in non-HDL-C in response to simvastatin and atorvastatin treatment (n=8,023)

| Variable | Effect estimate(95%CI) , P-value | | |
| --- | --- | --- | --- |
|  | Univariate analysis  (Model 1) | Model 2 | Model 3 |
| *CYP3A4* rs2740574 | 0.40(-0.15,0.94), 0.152 | 0.36(-0.17,0.90), 0.181 | 0.34(-0.11,0.78), 0.140 |
| Percentage of daily coverage | - | 0.26(0.24,0.29) P<0.001 | 0.21(0.18,0.23), <0.001 |
| Switching | - | -0.33(-0.47,-0.20), <0.001 | -0.26(-0.38,-0.14), <0.001 |
| Dose reduction | - | -0.05(-0.09,-0.003), 0.035 | -0.12(-0.16,-0.08), <0.001 |
| Sex | - | - | -0.003(-0.04,0.04)0.876 |
| Age | - | - | 0.01(0.01,0.014),<0.001 |
| BMI | - | - | -0.006(-0.009,-0.002),0.001 |
| Mean dose | - | - | 0.006(0.005,0.007),<0.001 |
| Duration of statin therapy | - | - | -0.05(-0.06,-0.03), <0.001 |
| Type 2 diabetes | - | - | 0.01(-0.001,0.02), 0.071 |
| History of MACE | - | - | -0.06(-0.11,-0.01), 0.029 |
| Non-HDL-C at baseline | - | - | 0.5(0.48,0.52), <0.001 |

Model 1: univariate effect, Model 2: features of statin intolerance, Model 3: demography ,features of statin intolerance and important comorbidities.

Supplementary Table 9. Effect of CYP3A5 (rs776746) with the absolute reduction in non-HDL-C in response to simvastatin and atorvastatin treatment (n=8,736)

| Variable | Effect estimate(95%CI) , P-value | | |
| --- | --- | --- | --- |
|  | Univariate analysis (Model 1) | Model 2 | Model 3 |
| *CYP3A5* rs776746 | 0.03(-0.04,0.10), 0.375 | 0.03(-0.03,0.10), 0.343 | 0.02(-0.04,0.07), 0.534 |
| Percentage of daily coverage | - | 0.27(0.24,0.30), <0.001 | 0.21(0.19,0.24), <0.001 |
| Switching | - | -0.33( -0.46,-0.21), <0.001 | -0.28(-0.39,-0.17), <0.001 |
| Dose reduction | - | -0.05(-0.10,-0.01), 0.014 | -0.13(-0.17,-0.1), <0.001 |
| Sex | - | - | -0.01(-0.05,0.03)0.561 |
| Age | - | - | 0.01(0.009,0.013),<0.001 |
| BMI | - | - | -0.004(-0.008,-0.001),0.12 |
| Mean dose | - | - | 0.006(0.005,0.007),<0.001 |
| Duration of statin therapy | - | - | -0.04(-0.06,-0.03), <0.001 |
| Type 2 diabetes | - | - | -0.10(-0.14,-0.05), <0.001 |
| History of MACE | - | - | -0.04(-0.102,-0.003), 0.036 |
| Non-HDL-C at baseline | - | - | 0.5(0.48,0.52), <0.001 |

Model 1: univariate effect, Model 2: features of statin intolerance, Model 3: demography features of statin intolerance and important comorbidities.

Supplementary Table 10. Effect of LILRB5 and ABCB1 two variant risk score with the absolute reduction in non-HDL-C to all statin treatment (n=8569)

| Variable | Effect estimate(95%CI) , P-value | | |
| --- | --- | --- | --- |
|  | Univariate analysis (Model 1) | Model 2 | Model 3 |
| LILRB5 rs12975366 (CC or TC) + ABCB1 rs1045642 (CC) vs.  LILRB5 rs12975366 (TT) + ABCB1 rs1045642 (CT or TT) | 0.14(0.07,0.20), <0.001 | 0.13(0.06,0.19), <0.001 | 0.1(0.05,0.16), <0.001 |
| Percentage of daily coverage | - | 0.26(0.24,0.29), <0.001 | 0.21(0.19,0.24),<0.001 |
| Switching | - | -0.31(-0.43,-0.18), <0.001 | -0.28(-0.39,-0.17), <0.001 |
| Dose reduction | - | -0.08(-0.12,-0.03), <0.001 | -0.16(-0.20,-0.12), <0.001 |
| Sex | - | - | -0.01(-0.05,0.03)0.759 |
| Age | - | - | 0.01(0.009,0.013),<0.001 |
| BMI | - | - | -0.004(-0.008,-0.001),0.015 |
| Mean dose | - | - | 0.006(0.005,0.007), <0.001 |
| Duration of statin therapy | - | - | -0.04(-0.05,-0.02), <0.001 |
| Diabetes | - | - | -0.09(-0.13,-0.04),<0.001 |
| History of MACE | - | - | -0.06(-0.11,0.01), 0.028 |
| Non-HDL-C at baseline | - | - | 0.5 (0.48,0.52), <0.001 |

Model 1: univariate effect, Model 2: features of statin intolerance, Model 3: demography, features of statin intolerance and important comorbidities.

Supplementary Table 11. Effect of LILRB5 and ABCB1 two-variant risk score with the absolute reduction in non-HDL-C to simvastatin treatment (n=6411)

| Variable | Effect estimate(95%CI) , P-value | | |
| --- | --- | --- | --- |
|  | Univariate analysis  (Model 1) | Model 2 | Model 3 |
| LILRB5 rs12975366 (CC or TC) + ABCB1 rs1045642 (CC) vs.  LILRB5 rs12975366 (TT) + ABCB1 rs1045642 (CT or TT ) | 0.16(0.09,0.23), <0.001 | 0.16(0.09,0.23),<0.001 | 0.12 (0.06,0.19),<0.001 |
| Percentage daily coverage | - | 0.29(0.26,0.32), <0.001 | 0.25(0.22,0.29),<0.001 |
| Switching | - | -0.27(-0.41,-0.14), <0.001 | -0.27(-0.39,-0.14), <0.001 |
| Dose reduction | - | -0.07(-0.11,0.02), 0.008 | -0.12(-0.16,-0.08), <0.001 |
| Sex | - | - | -0.003(-0.05,0.04)0.891 |
| Age | - | - | 0.01(0.011,0.015),<0.001 |
| BMI | - | - | -0.004(-0.008,-0.0004),0.03 |
| Mean dose | - | - | 0.012(0.01,0.014), <0.001 |
| Duration of statin therapy | - | - | -0.03(-0.05,-0.02), <0.001 |
| Type 2 diabetes | - | - | -0.06(-0.11,-0.01),0.022 |
| History of MACE | - | - | -0.04(-0.10,0.01), 0.140 |
| Non-HDL-C at baseline | - | - | 0.48 (0.46,0.52), <0.001 |

Model 1: univariate effect, Model 2: features of statin intolerance, Model 3: demography features of statin intolerance and important comorbidities.
